# LHFPL5 is a key element in force transmission from the tip link to the hair cell mechanotransducer channel

**DOI:** 10.1073/pnas.2318270121

**Published:** 2024-01-09

**Authors:** Maryline Beurg, Evan Travis Schwalbach, Robert Fettiplace

**Affiliations:** ^a^Department of Neuroscience, University of Wisconsin School of Medicine and Public Health, Madison, WI 53706

**Keywords:** cochlea, MET channel, TMC1, LHFPL5, cholesterol

## Abstract

During auditory transduction, sound-evoked vibrations of the hair cell stereociliary bundles open mechanotransducer (MET) ion channels via tip links extending from one stereocilium to its neighbor. How tension in the tip link is delivered to the channel is not fully understood. The MET channel comprises a pore-forming subunit, transmembrane channel-like protein (TMC1 or TMC2), aided by several accessory proteins, including LHFPL5 (lipoma HMGIC fusion partner-like 5). We investigated the role of LHFPL5 in transduction by comparing MET channel activation in outer hair cells of *Lhfpl5−/−* knockout mice with those in *Lhfpl5+/−* heterozygotes. The 10 to 90 percent working range of transduction in *Tmc1+/+; Lhfpl5+/−* was 52 nm, from which the single-channel gating force, Z, was evaluated as 0.34 pN. However, in *Tmc1+/+; Lhfpl5−/−* mice, the working range increased to 123 nm and Z more than halved to 0.13 pN, indicating reduced sensitivity. Tip link tension is thought to activate the channel via a gating spring, whose stiffness is inferred from the stiffness change on tip link destruction. The gating stiffness was ~40 percent of the total bundle stiffness in wild type but was virtually abolished in *Lhfpl5−/−,* implicating LHFPL5 as a principal component of the gating spring. The mutation *Tmc1* p.D569N reduced the LHFPL5 immunolabeling in the stereocilia and like *Lhfpl5−/−* doubled the MET working range, but other deafness mutations had no effect on the dynamic range. We conclude that tip-link tension is transmitted to the channel primarily via LHFPL5; residual activation without LHFPL5 may occur by direct interaction between PCDH15 and TMC1.

## Significance

The mechano-electrical transducer (MET) channel in cochlear hair cells is opened by tension in tip links joining adjacent stereocilia.Force may be transmitted directly from PCDH15 at the lower end of the tip link to TMC1 (the pore-forming subunit) or indirectly via the accessory subunit LHFPL5. We investigated the role of LHFPL5 by measuring MET channel activation curves and hair bundle stiffness in OHCs of wild-type mice and *Lhfpl5* mutants. Transducer sensitivity and single-channel gating stiffness were much reduced in *Lhfpl5* knockouts, though it was still possible to activate MET currents. Similar effects were seen in *Tmc1* p.D569N mutants which reduce LHFPL5 expression at the channel. These results endorse LHFPL5 as the primary conduit of force to the channel.

Mechanical sensitivity is a primitive sensation present in both prokaryotic and eukaryotic organisms and is served by specialized mechanoreceptor ion channels in the plasma membrane of sensory cells for monitoring tissue deformation. Mechanical sensation is mediated by a disparate array of unrelated ion channel proteins including MscL in bacteria, OSCA1.2 in plants like Arabidopsis, MEC-4 in worms, NOMPC in flies, and PIEZO1/2 in vertebrates (1, 2). These mechanically sensitive ion channels have evolved with traits of speed and sensitivity appropriate for the organism, but the majority can be directly activated by tension in the lipid bilayer (3, 4). Indeed, several such proteins, including MscL and PIEZO1, when inserted into a reduced system composed only of lipid vesicles, can be gated by mechanical force transmitted through the lipid bilayer; this has been referred to as “force-from-lipid” (4, 5).

The mechano-electrical transducer (MET) channel of cochlear hair cells is probably the most specialized mechanoreceptor, responsible for detecting and transducing sound stimuli into electrical signals. This channel is unusual in being rapidly activated, capable of responding cycle-by-cycle to 100 kHz sounds, and ultrasensitive, resolving displacements less than a nanometer near the limits set by Brownian motion (6–9). Unlike other mechanoreceptors, the hair cell MET channel may be gated primarily by force delivered through tethers from the extracellular matrix and cytoskeleton. Sound transmitted to the cochlea evokes displacements of the hair-cell stereociliary bundles, the vibrations of which are funneled to the MET channel through force in extracellular tip links (7). These tip links extend from the side wall of one stereocilium to the top of its neighbor where the channel is located (10). How tension in the tip link modulated by sound stimuli is delivered to the channel is not fully understood.

The lower end of the tip link, formed by dimers of protocadherin-15 (PCDH15), is anchored to the top of the stereocilium in an electron-dense region likely to represent the proteins of the MET channel complex. The multiprotein complex is thought to comprise a pore-forming subunit, transmembrane channel-like protein (TMC1 or TMC2) (11) aided by several accessory proteins, including LHFPL5 (lipoma HMGIC fusion partner-like 5), TMIE (transmembrane inner ear), and CIB2 (Ca^2+^ and integrin binding protein) that connects to the actin cytoskeleton (12, 13). Thus, the present view is that the intracellular tether is CIB2 (14–16) and the extracellular tether is PCDH15 whose lower end (C terminus) interacts with both TMC1 and LHFPL5. TMC1 is coimmunoprecipitated by PCDH15 when expressed in HEK cells (17), and results indicate that PCDH15 binds TMC1 and zebrafish TMC orthologs (18). In both biochemical and cryo-EM structural studies, the transmembrane and cytoplasmic domains of PCDH15 also bind to LHFPL5 (17, 19). The observations are consistent with the hypothesis that tension in the tip link can be transmitted to the channel through the interaction of PCDH15 with LHFPL5, which is then coupled to TMC1 (20–22). The experimental aim was to test this hypothesis of force transmission by investigating the contribution of LHFPL5 to transduction in cochlear hair cells of *Lhfpl5* knockout mice. Mutations affecting LHFPL5 lead to autosomal recessive nonsyndromic hearing loss (DFNB67) in humans (23), and deafness and vestibular dysfunction in mice (24). Early neonatal MET currents in *Lhfpl5−/−* are present but smaller than wild type (17). Transduction was assessed from MET current activation curves, which provide a measure of the single-channel gating force (25, 26), and from measurements of hair bundle stiffness after tip links destruction to derive the single-channel gating stiffness (27, 28). A potential role for LHFPL5 in force transmission was suggested in previous work (21, 29).

## Methods

### Mouse Mutants.

*Tmc1* p.D528N and *Tmc1* p.D569N mice were made by Horizon Labs Inc. (Saint Louis, MO, now owned by Inotiv, West Lafayette, IN) using CRISPR/Cas9 technology (30, 31). *Tmc1* p.M412K (*Beethoven*) mice were obtained from Karen Steel (Kings College, London) and Walter Marcotti (Sheffield University, UK). The *Lhfpl5−/−* mouse strain was the B6.129-*Lhfpl5^tm1Kjn^*/Kjn (Jackson Labs, strain 005434). The mutations were bred on an FVB background in wild type (*Tmc1+/+; Tmc2+/+*) and on a *Tmc2−/−* background. *Tmc2−/−* mice (B6.129S5-*Tmc2^tm1Lex^*/Mmucd) were obtained from the Mutant Mouse Regional Resource Center (University of California, Davis, CA). TMC2 is expressed early on at postnatal day (P)2 in apical cochlear hair cells but is largely replaced by TMC1 by P6 (32). Neonatal mice up to P7 were killed by decapitation according to the animal protocol approved by the Institutional Animal Care and Use Committee at the University of Wisconsin-Madison. Both male and female mice were used in approximately equal proportions and were kept on a 12 h light/12 h dark cycle with food and water ad libitum.

### Hair Cell Recording and Stimulation.

MET currents were recorded from outer hair cells (OHCs) in isolated organs of Corti of mice between P2 and P7 as previously documented (30, 33). Unless otherwise noted, hair-cell recordings were made at an apical cochlear location at *d* = 0.2 to 0.3, where *d* is the distance along the basilar membrane from the apex divided by its total length. A few recordings were made at a basal location at *d* = 0.8 The recording chamber was mounted on the stage of a Leica DMLFS top-focusing microscope and viewed with 40× (numerical aperture, NA, = 0.8) objective and a 2× optivar. The chamber was perfused with saline (composition in mM): 152 NaCl, 6 KCl, 1.5 CaCl_2_, 2 Na-pyruvate, 8 D-glucose, and 10 Na-HEPES, pH 7.4. Patch electrodes were filled with (in mM): 138 CsCl, 3.5 MgCl_2_, 5 Na_2_ATP, 0.5 Na_2_GTP, 10 Tris phosphocreatine, 1 EGTA (ethylene glycol-bis(β-aminoethyl ether)-*N*,*N*,*N*′,*N*′-tetraacetic acid), and 10 Cs-HEPES, pH 7.2, and and they were connected to an Axopatch 200B amplifier. The series resistances of patch electrodes with 60 percent compensation applied were at best 3 MΩ, which, with a ~5 pF cell capacitance, gave a recording time constant of 15 µs. MET currents were smoothed with an eight-pole filter at 3 kHz. Methyl-β-cyclodextrin to deplete membrane cholesterol (Cayman Chemical Co; Ann Arbor, MI), water-soluble cholesterol (C4951; Sigma-Aldrich, St. Louis, MO), and the Na^+^ salt of cis 4,7,10,13,16,19 docosahexanoic acid (D8768; Sigma-Aldrich, St. Louis, MO) were perfused in the extracellular saline. All experiments were performed at room temperature ~23 °C. Results are quoted as mean ± 1 SD, for N, the number of OHCs (usually one cell per preparation), and statistical significance was assessed with a two-tailed Student *t* test.

Stereociliary bundles were stimulated mostly with a fluid jet or occasionally with a glass probe driven by a piezoactuator. The amplitudes of hair bundle deflections were calibrated by projecting the bundle image onto a pair of photodiodes and measuring the change in photocurrent (34, 35). The relationship between the MET current, I, and bundle displacement, X, was fitted with a Boltzmann equation: I = I_MAX_ /(1 + exp((X_O_ − X)/X_S_), where I_MAX_ is the maximum current, X_O_ the half-saturating displacement and X_S_ the slope factor. The 10 to 90 percent working range of transduction (WR) is 4.4*X_S_ and, according to the gating spring model, WR = 4k_B_T/Z, where k_B_ is the Boltzmann constant, T is temperature and Z is the single-channel gating force. Z provides a measure of gating sensitivity (26). I-X relationships were determined from the second and third cycles of the current response to sinusoidal fluid jet stimulation.

**Fig. 1.**
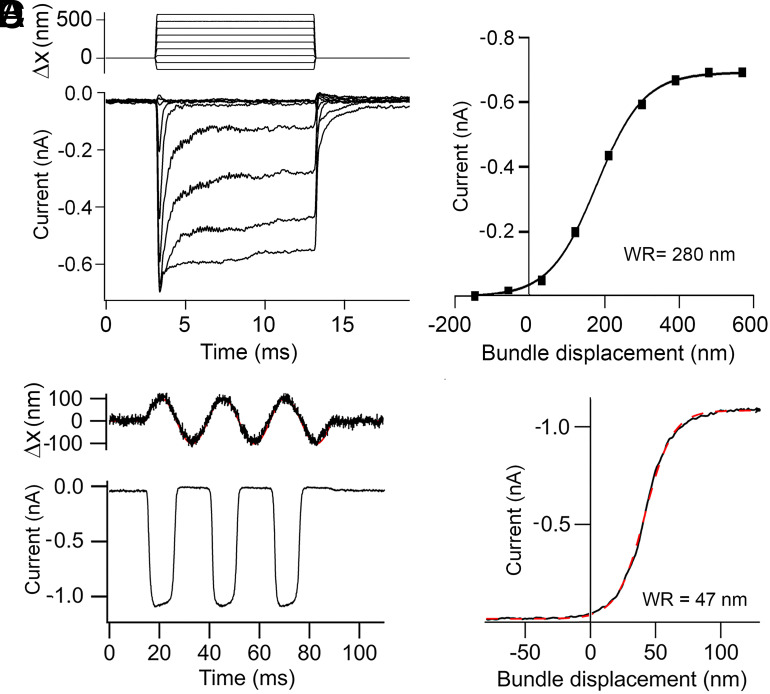
The dynamic range of MET current activation depends on the method of stimulation. (*A*) MET currents in apical OHC in response to step stimuli applied to the hair bundle with a glass probe driven by a piezoelectric actuator. Imposed displacements are shown above. (*B*) Relationship between peak MET current and probe displacement (filled squares) for records in *A*. The smooth curve is a fit with a single Boltzmann equation (*Methods*) with I_MAX_ = 0.68 nA, half-saturation X_O_ = 170 nm, and slope X_S_ = 64 nm. 10 to 90 percent working range, WR = 280 nm. (*C*) MET currents evoked by a sinusoidal fluid jet stimulus; bundle displacements measured with photodiode method depicted in noisy trace above. (*D*) The black curve is activation relationship from records in *C* fit with a single Boltzmann equation (red dashed line) with I_MAX_ = 1.07 nA, half-saturation X_O_ = 40 nm, and slope X_S_ = 10.6 nm. 10 to 90 percent working range, WR = 47 nm. Holding potential −84 mV. Apical OHCs in P5 *Tmc1+/+; Tmc2−/−* mice.

WR, the dynamic range of mechanotransduction, is a crucial variable in the experiments and there is considerable (up to 10-fold) spread in the literature values, which may partly reflect the mode of bundle stimulation (28, 36, 37). For determining WR, we used a fluid jet and directly measured bundle motion, but others in their assessments of WR used glass probes attached to the bundle (21, 38). The latter are glass pipettes fire-polished, so their ends fit into the “W-shape” of the OHC bundle (39). Such glass probes are likely to produce uneven stimulation (36) and may substantially overestimate WR, which will confound the effects of mutants on the gating spring. Fig. 1 compares the two types of stimulation: the glass probe imposed a step stimulus (Fig. 1*A*) and gave an I-X relationship with mean WR of 297 ± 39 nm (N = 5; I_MAX_ = 0.75 ± 0.1 nA); the fluid jet I-X activation curve in the present experiments was derived from sinusoidal stimuli and gave a mean WR of 53 ± 11 nm (N = 17; I_MAX_ = 0.98 ± 0.2 nA). Both sets of experiments were performed on apical OHCs of P5-P7 *Tmc1+/+; Tmc2+/+* mice. The fluid jet WR values are close to those inferred from in vivo experiments (7) for which WR values of 30 to 100 nm for OHC transduction were derived from the range of basilar membrane movements over which the cochlear nonlinearity between sound pressure and displacement were observed. In these in vivo experiments, OHC bundles would be stimulated physiologically via insertion of the tallest stereociliary row into the tectorial membrane (40, 41), and the dynamic range reflects an increase in receptor current from a small to a saturating amplitude.

### Isolated Outer Hair Cells.

Apical or basal turns of the organ of Corti were dissected in Hanks’ Balanced Salt Solution (HBSS) from age P4 (base) or P5-P7 (apex) mice. Organs of Corti were incubated in 50 µg/mL Collagenase IV (Sigma-Aldrich; St Louis MO) for 15 min, and enzymatic activity was then terminated by washing in 50 mg/mL BSA for ~2 min. Mechanical dissociation of hair cells was performed in HBSS by trituration with a 200 µL Eppendorf pipette with a cut tip in a glass-bottom dish coated with CellTak (Corning NY). Hair cells were allowed to settle for 10 min and then observed on an inverted Zeiss Axiovert 10 microscope with a 63× oil immersion Plan-achromat objective. Hair bundle length was measured from the root to the tip of the longest stereocilia.

### Hair Bundle Stiffness.

Hair bundle stiffness was determined by calibrating the force generated by the fluid jet, which was formed from a glass pipette, internal tip diameter ~10 µm, filled with extracellular saline, and placed on the neural aspect of the hair bundle. The velocity of the fluid stream at the hair bundle was calculated using Stokes Law, calibrated from the displacement of a flexible glass fiber inserted into the fluid stream at the same distance (9.9 ± 0.5 µm) from the mouth of the fluid jet pipette as was the bundle (28, 42). The viscous force, F, on a sphere radius R moving at velocity V in a fluid stream is given:

[1] F= 6πRηV,

where η is the saline viscosity, 10^−3^ N m^−2^ s. Neither the hair bundle nor the glass fiber was spherical, but it is possible to calculate for each structure an effective (spheroidal) radius R_EFF_. For the apical OHC hair bundle, an effective bundle radius R_HB_ of 2.3 µm was used, about half the total bundle height, calculated from equation 2 in ref. 42. The flexible glass fiber was 1 µm in diameter, from which an effective radius R_F_ = 3 µm was inferred as described in ref. 28. In addition, according to calculations (28), the force on the hair bundle produced by the fluid jet was about 30 percent more effective (larger) than that on the fiber. The calibration procedure, performed on each fluid jet pipette, was to measure the deflection of the fiber ΔX_F_ for a given stimulus ΔS_F_ to the fluid jet, calculate the force F_F_ on the fiber (K_F_*ΔX_F_), and apply (Eq. **1**) to determine the velocity V. This value of V was then inserted back into Eq. **1** to infer the force on the hair bundle F_HB_ using the appropriate effective radius R_HB_, and measure bundle displacement ΔX_HB_. Hair bundle stiffness K_HB_ is then given by F_HB_/ΔX_HB_. In practice, many of the constants including the R_EFF_ values cancel out, so the bundle stiffness is

[2] $$K_{\text{HB}}\;=\;\frac{K_{F}\;\times \;\Delta X_{F}\;\times \;\Delta S_{\text{HB}}}{\Delta X_{\text{HB}}\;\times \;\Delta S_{F},}$$

The stiffness of the calibrating glass fiber, determined by measuring the vertical deflection caused by hanging beads of methyl methacrylate on the fiber tip (27, 34), was 1 mN/m.

### Immunolabeling.

Cochleae were fixed in 4% paraformaldehyde for 30 min at room temperature. Fixed tissue was incubated in goat serum, a blocking agent, for 1 h and immunolabeled with the appropriate primary antibody, then a secondary Alexa Fluor488 antibody (1:400) and Alexa-568 phalloidin (1:500, 1.5 h). Immunolabeled cochleae were mounted with Fluoromount G and viewed on a Nikon A1 confocal microscope with a 60× objective (NA = 1.4) or 100× objective (NA = 1.45), and the fluorescence intensity was measured with ImageJ (Fiji) software. LHFPL5 localization was assessed with a polyclonal anti-LHFPL5 antibody (Aviva, San Diego; OAAB00424). To test for the presence of a second isoform, LHFPL4, cochleae were immunolabeled with polyclonal anti-LHFPL4 antibodies (Sigma SAB2109206; 1:100), made against the C terminus in a region dissimilar to LHFPL5, and then treated with anti-rabbit Alexa Fluor-568 secondary antibody (1:400). Relative tip link number was assessed by labeling PCDH15-CD2 (43) with a polyclonal antibody against the peptide sequence CSEGEKARKNIVLARRRP (Bethyl Fortis Life Sciences, Montgomery TX).

A positive control for the anti-LHFPL4 antibody was obtained by labeling GABA_A_ receptors, which are thought to interact with LHFPL4 (44, 45). GABA_A_ receptors in the inner plexiform layer of mouse retina were used for this control. P29 mice were anesthetized with isoflurane using procedures approved by the Institutional Animal Care and Use Committee at the University of Wisconsin-Madison. They were decapitated, the eyes excised, and after removal of the vitreous, the retina was isolated and fixed for 15 min in 4% paraformaldehyde. The retinal whole mount was immunolabeled with an antibody to LHFPL4, followed by an Alexa Fluor-488 secondary antibody (1.5 h), and then a primary against GABA_A_-γ2 (Synaptic Systems 224 003, Göttingen, Germany; 1:100, 2 h, RT), followed by an Alexa Fluor-568 secondary antibody.

## Results

### *Lhfpl5* Knockout Broadens the I-X Relationship.

**Fig. 2.**
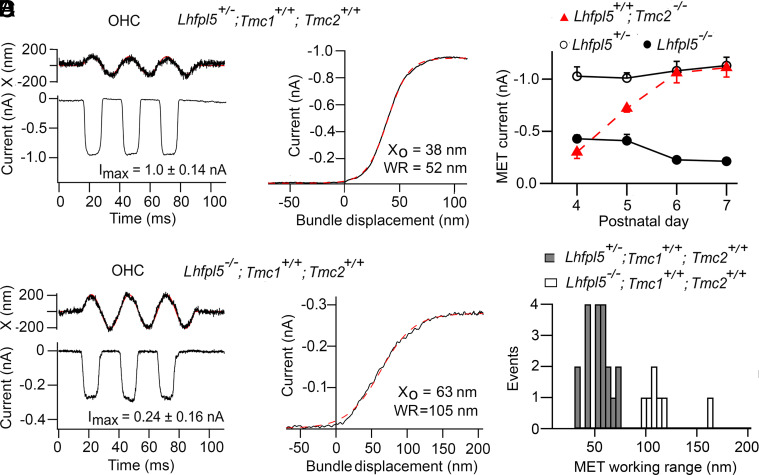
Effects of *Lhfpl5* knockouts on MET currents in apical OHCs. (*A*) MET current in P5 OHC of *Lhfpl5+/−* heterozygote, elicited with a sinusoidal fluid jet; bundle motion is noisy trace above. On the right is the activation relation, fit with a single Boltzmann equation (red dashed line) with I_MAX_ = 0.93 nA, half-saturation X_O_ = 38 nm, and slope X_S_ = 11.8 nm. 10 to 90 percent working range, WR = 52 nm. (*B*) MET current in P5 OHC of *Lhfpl5−/−* homozygote, bundle motion is noisy trace above. On the right is the activation relation, fit with a single Boltzmann equation (red dashed line) with I_MAX_ = 0.28 nA, half-saturation X_O_ = 63 nm, and slope X_S_ = 24 nm. 10 to 90 percent working range, WR = 105 nm. (*C*) Time course of neonatal development of the mean MET current amplitude in *Lhfpl5* mutants. Open circles, *Lhfpl5+/−* heterozygotes on *Tmc1+/+; Tmc2+/+* background; numbers of cells, 8, 14, 8, and 4. Filled circles, *Lhfpl5−/−* homozygotes on *Tmc1+/+; Tmc2+/+* background, numbers of cells, 11, 10, 13, and 4. Red triangles, time course of MET current development in *Tmc1+/+; Tmc2−/−,* numbers of cells, 7, 5, 6, and 3, Results indicate that the current is carried largely by TMC2-containing channels at P4, but by TMC1 after P6, when TMC2 is downregulated. Thus, *Lhfpl5* knockout reduces TMC2 current (P4) by 58 percent and TMC1 current (P7) by 80 percent. (*D*) Distribution of working ranges (WR) for recordings from Lhfpl5+/− and Lhfpl5−/− in apical OHCs from P4 to P6 mice.

Two control mice were studied, wild type (*Tmc1+/+;Tmc2+/+*) and TMC1 (*Tmc1+/+;Tmc2−/−*), the latter being the genotype in adult mice (46). Mutants was tested without or with knockout of *Lhfpl5*, and in each case the relation between MET current and bundle displacement (the I-X curve) was determined. The I-X curve for the wild type, *Lhfpl5+/−* heterozygote, had a WR of 52 ± 10 nm (N = 17) with a maximum current of 1.00 ± 0.14 nA. For the *Lhfpl5−/−* homozygote, there was a reduction in the maximum current and a broadening of the I-X curve, to give WR = 123 ± 12 nm (N = 10) (Fig. 2 *A* and *B*). *Lhfpl5* knockout reduces the maximum current at both P4, when the TMC2 isoform is the predominant channel protein, and at P7 when most channels are composed of TMC1, indicating that LHFPL5 interacts with both TMC isoforms. The relative contributions of the two isoforms are evident by comparing development of the MET currents in wild type, *Tmc1+/+; Tmc2+/+*, with development in *Tmc1+/+; Tmc2−/−* (Fig. 2*C*). The reduction in MET current in *Lhfpl5−/−* is larger at P7 when the channels are composed predominantly of the TMC1 isoform (80 percent) than at P4 with TMC2 (60 percent), suggesting a stronger interaction with TMC1. When only TMC1 was present, the control WR was 44 ± 6 nm (N = 7). However, in the absence of LHFPL5, the maximum currents were smaller, but it was still possible to measure a WR of 125 ± 18 nm (N = 5) (Fig. 2 *C* and *D*). In both wild-type and *Tmc2−/−* mutants, there was also a positive shift in the I-X curve in the *Lhfpl5* knockout compared to the control. In the wild type, the half-activation, X_O_, was 39 ± 10 nm (N = 17) in *Lhfpl5+/−*, whereas it was 123 ± 37 nm (N = 10) in *Lhfpl5−/−*, showing an 84 nm positive shift in the absence of LHFPL5. With *Tmc2−/−* mice, X_O_ was 153 ± 13 nm in *Lhfpl5−/−* (N = 4), a significant positive shift of 95 nm (*t* test*, P* = 0.005). The broadening and translation of the I-X curves indicate a decrease in mechanical sensitivity of the MET channel in the *Lhfpl5* knockout.

The WR values were used to calculate Z, the single-channel gating force using the relation Z = 4k_B_T/WR (26). For *Tmc1+/+; Tmc2+/+; Lhfpl5+/−* and *Tmc1+/+; Tmc2−/−*; *Lhfpl5+/−* mice, the single-channel gating force was 306 ± 64 fN (N = 10) and 344 ± 37 fN (N = 5) respectively, there being no difference between the two controls (*t* test*, P* = 0.54). In both cases, the single-channel gating forces in the absence of LHFPL5 were reduced to 132 ± 13 fN and 130 ± 19 fN, respectively, the difference between the two again being insignificant. The reduction in the gating force confirms a decrease in mechanical sensitivity without LHFPL5 in both isoforms.

**Fig. 3.**
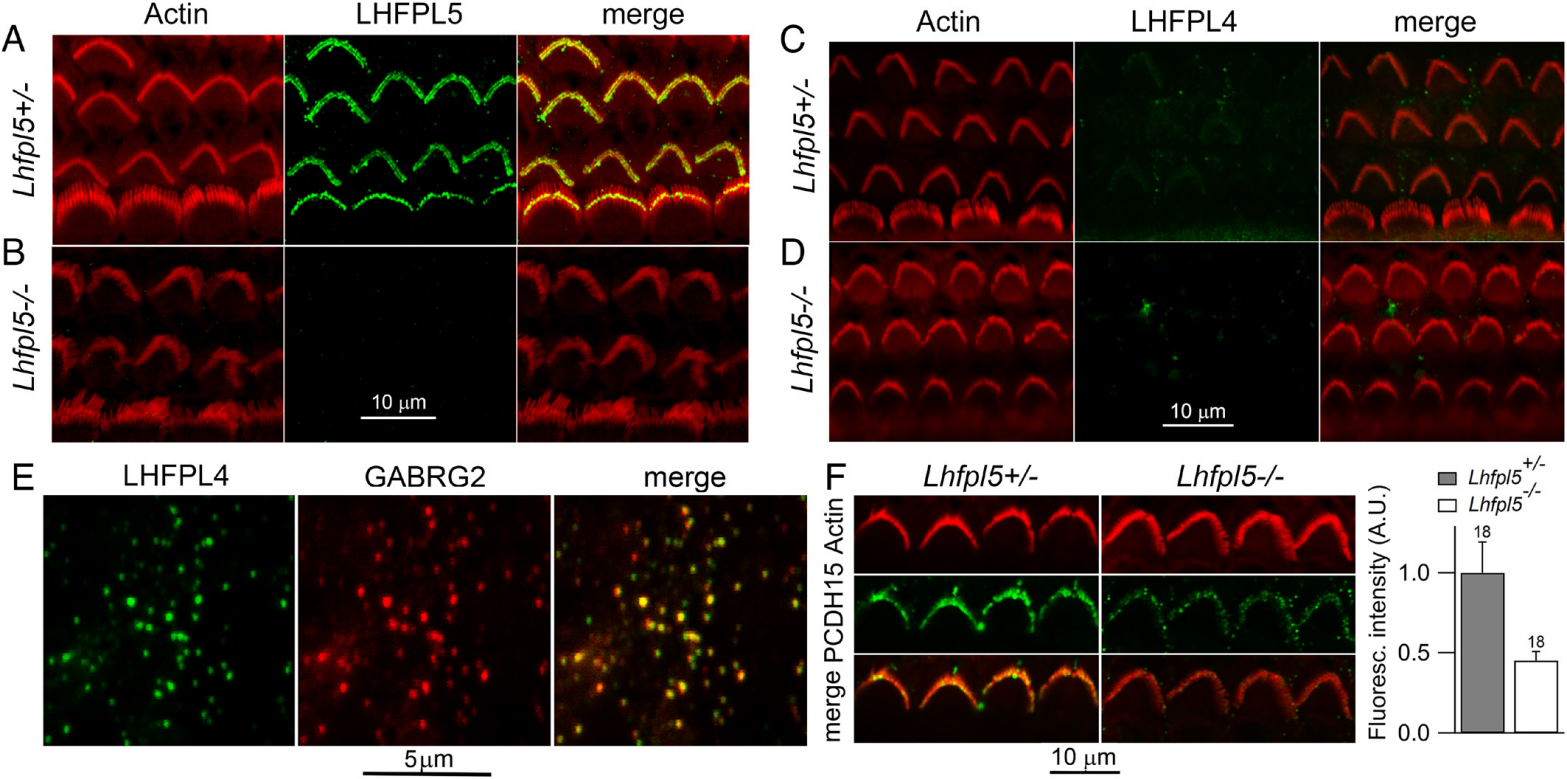
Immunolabeling for LHFPL5 and LHFPL4. (*A*) Labeling for actin with phalloidin, LHFPL5, and merge of two in *Lhfpl5+/−* P6 mouse. (*B*) The same labeling in *Lhfpl5−/−* showing loss of LHFPL5 label. (*C*) Labeling for actin with phalloidin, LHFPL4 (Sigma antibody), and merge of two in *Lhfpl5+/−* P6 mouse. (*D*) The same labeling in Lhfpl5−/−. Note that no LHFPL4 label was visible in hair bundles of either *Lhfpl5* heterozygote or homozygote. (*E*) Positive control for the LHFPL4 antibody, labeling inhibitory GABAergic synapses in retinal whole mounts, which were colabeled with GABA_A_ gamma-2 subunit (GABGR2). (*F*) Labeling for the PCDH15-CD2 antibody to assess tip link numbers; right, a 55 percent reduction in fluorescence labeling (difference significant, 2 × 10^−8^) in *Lhfpl5* knockouts of P5 mice.

There are four LHFPL isoforms present in the cochlea in the age range P4 to P7 according to the SHIELD database (47). Of these, significant amounts occur in the hair cells only for LHFPL4 and LHFPL5, the former isoform being expressed at one-tenth that of LHFPL5. There was pronounced immunolabeling for LHFPL5 in the *Lhfpl5* heterozygote but it disappeared in the *Lhfpl5* knockout (Fig. 3 *A* and *B*) corroborating the electrophysiological results. We also examined whether LHFPL4 was present in the hair bundles in the presence or absence of the main isoform LHFPL5. Lack of labeling was found in both *Lhfpl5* heterozygote and *Lhfpl5* knockout (Fig. 3 *C* and *D*) suggesting little or no presence of LHFPL4 in the bundles. The result bolsters the finding that rescue of the MET current in cultured cochleae from *Lhfpl5−/−* mice took place only after injectoporation of message for LHFPL5 but none of the other isoforms, including LHFPL4 (21). A control to validate the LHFPL4 antibody was obtained by colabeling GABA_A_ receptors, which have been reported to interact with LHFPL4 to promote receptor clustering in the hippocampus (44). LHFPL4 is also concentrated in the retina (Human Protein Atlas), and we found colocalization of labels for LHFPL4 and for the GABA_A_-gamma-2 subunit (Pearson’s R-value of 0.8, where 1.0 is perfect overlap) in the inner plexiform layer of mouse retinal whole mounts (Fig. 3*E*), supporting use of the LHFPL4 antibody. The *Lhfpl5* knockout is associated with reduction in the number of tip links (22), the extent of which we estimated by labeling for PCDH15-CD2 (Fig. 3*F*). The relative immunofluorescence (knockout/control) was 0.45 ± 0.08 (N = 18 bundles).

### *Lhfpl5* Knockout Decreases Gating Stiffness.

**Fig. 4.**
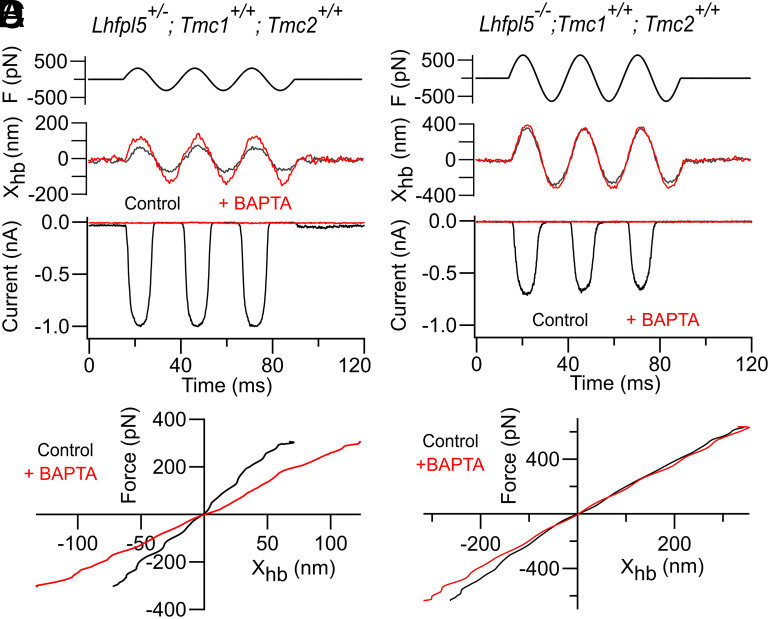
Effects of *Lhfpl5* knockout on MET channel gating stiffness. (*A*) MET currents in *Lhfpl5+/−* heterozygote evoked by sinusoidal fluid jets before (black traces) and after (red traces) severing the tip links with BAPTA-containing saline. *Top*, driving force F derived as described in methods, middle hair bundle motion X_hb_, *Bottom* MET currents. BAPTA abolishes current and increases bundle displacements for identical force. (*B*) Force-displacement relationship in *Lhfpl5+/−* heterozygote; BAPTA reduces the hair bundle stiffness from 5.1 mN/m to 2.7 mN/m, the difference being the gating stiffness. (*C*) MET currents in *Lhfpl5−/−* for sinusoidal fluid jet stimulation, before (black traces) and after (red traces) severing the tip links with BAPTA-containing saline. Note that BAPTA abolishes current but has minimal effect on bundle displacement. (*D*) Force–displacement relationship in *Lhfpl5* homozygote; BAPTA has little effect on hair bundle stiffness reducing at most from 2.0 mN/m to 1.9 mN/m, indicating small gating stiffness. In *C* and *D*, the photodiode signals were low-pass filtered at 1 kHz to obtain smooth force-displacement plots. Holding potential −84 mV. Apical OHCs in P5 *Tmc1+/+; Tmc2+/+* mice.

The prevailing view of hair cell transduction is that tension in the tip links opens MET channels (48), acting via a gating spring (25). The stiffness of the gating spring can be derived from the change in hair bundle stiffness on severing the tip links. In its simplest (approximate) form, the hair bundle stiffness, K_HB_, is the parallel combination of the pivot stiffness, K_SP_, at the stereociliary ankles (where the stereocilia flex during bundle deflection), and the stiffness, K_GS_, of the gating spring: thus, K_HB_ = K_SP_ + K_GS_. This formulation was used to investigate the contribution of LHFPL5 to gating stiffness. In control apical OHCs with heterozygotic *Lhfpl5+/−*, the total stiffness of the hair bundle was determined as 5.1 ± 2.1 mN/m (mean ± SD; N = 11), similar to the measurements of others on apical OHCs of mice (42) and rats (27, 28). After destroying the tip links by perfusing with saline containing 5 mM BAPTA, hair bundle stiffness was reduced by about a third (Fig. 4*A*) to 3.2 ± 1.1 mN/m (N = 7), the stiffness difference being significant in those seven cells (paired *t* test*, P* = 0.0005). The mean ratio K_GS_/K_HB_ was 0.38 ± 0.04 (N = 7), which was very similar to our prior measurements in rat OHCs (K_GS_/K_HB_ = 0.39 ± 0.04, N = 5) (27), but that ratio was smaller than 0.5 determined by others on rat apical OHCs (28). Force-displacement relations were linear with and without BAPTA treatment (Fig. 4*B*) as found in rat cochlear hair cells (28), with no hint of the nonlinearity reported in nonmammalian hair cells (49). When the same experiment was performed on *Lhfpl5−/−* homozygotes, hair bundle stiffness was 5.5 ± 2.3 mN/m (N = 7), not significantly different from that of the control *Lhfpl5+/*−. However, treatment with BAPTA saline now had a minimal effect on bundle stiffness (Fig. 4 *C* and *D*), and a mean ratio K_GS_/K_HB_ = 0.04 ± 0.02 (N = 5) was calculated. This value is small but significantly greater than zero (*t* test, *P* = 0.029). In apical OHCs of *Lhfpl5−/−* mice, the maximum current was 0.47 ± 0.17 nA (N = 5).

The analysis can be extended using the stiffness measurements to infer the single-channel gating stiffness *k*_GS_ (25, 26, 28, 50). *k*_GS_ depends on the number of tip links, N_TL_, and a geometrical factor γ, determined largely by the ratio of the stereociliary separation at the ankles, s, divided by bundle height h: (γ = s/h) (51):

[3] $$K_{\text{GS}}=\kappa_{\text{GS}}\times N_{\text{TL}}\times \gamma^{2}.$$

The stereociliary separation “s” was obtained from scanning electron micrographs of apical OHCs of wild type (29, 52) as 0.58 ± 0.1 µm (N = 22), after correction for shrinkage during fixation, and bundle height was measured on dissociated OHCs as 4.1 ± 0.3 µm (N = 18); bundle height and stereociliary separation taken together give γ = 0.14 (Table 1). Using N_TL_ = 47 (29), a single-channel gating stiffness, κ_GS_, of 2.2 ± 0.9 mN/m was calculated in OHCs of *Lhfpl5+/−* heterozygotes having a maximum MET current of 1.04 ± 0.14 nA (N = 5). With the *Lhfpl5−/−* knockouts, the number of tip links, N_TL_, was reduced to 0.45 (Fig. 3*F*), and the geometrical factor, γ, increased to 0.18 due to a reduction in bundle height with the same value for s (52) (Table 1). Applying Eq. **3**, the single-channel gating stiffness in *Lhfpl5* knockouts was 0.20 ± 0.1 mN/m (N = 5). Thus, the single-channel gating stiffness has been reduced 12-fold on loss of LHFPL5. Nevertheless, the MET channel can still be gated without LHFPL5, but alternative routes for force transmission must supply significantly smaller gating stiffness.

**Table 1.** Hair bundle and MET channel properties in *Tmc1* and *Lhfpl5* mutants

Mutant	I_MAX_ (nA)	H_B_, (µm)	K_GS_ (mN/m)	N_TL_	γ	κ_GS_ (mN/m)
WT *Lhfpl5+/−* (apex)	1.00 ± 0.14 (17)	4.1 ± 0.3 (18)	2.0 ± 1.1 (7)	47	0.14	2.2 ± 1.2
WT *Lhfpl5−/−* (apex)	0.48 ± 0.11 (4)	3.3 ± 0.4 (20)	0.13 ± 0.08 (5)	21	0.18	0.2 ± 0.12
WT *Lhfpl5+/−* (base)	1.29 ± 0.14 (4)	2.3 ± 0.2 (9)	6.3 ± 1.8 (4)	70	0.22	1.9 ± 0.5
*Tmc1* p.D569N (apex)	0.39 ± 0.1 (6)	2.8 ± 0.2 (11)	0.22 ± 0.2 (5)	20	0.20	0.28 ± 0.25

I_MAX_, maximum MET current, H_B_, height of tallest stereociliary row in hair bundle, K_GS_ stiffness of bundle gating springs; N_TL_, number of tip links, γ stimulus amplification factor; single-channel gating stiffness, κ_GS_ = K_GS_/(N_TL_*γ^2^). *Tmc1 p.*D569N is a mutation that interferes with LHFPL5 binding to TMC1.

A few measurements were made on basal OHCs tuned to high frequencies. The activation curves for the MET current had a WR of 34 ± 2 nm, yielding Z = 480 ± 28 fN. The hair bundle stiffness of basal OHCs was 18.1 ± 4.4 mN/m, several-fold larger than apical OHCs and the gating stiffness, inferred from BAPTA treatment, was to 6.3 ± 1.8 mN/m (N = 4). These tonotopic effects have been previously observed in rat OHCs (28). They may be partly attributable to an increase in N_TL_ to 70 (29) and an increase in γ to 0.22 because of a smaller bundle height. Applying these values in Eq. **3**, the single-channel gating stiffness for basal OHCs was calculated as 1.9 ± 0.5 mN/m; this value is not different from that for apical OHCs of 2.2 mN/m (*t* test*, P* = 0.69), suggesting that the gating stiffness is identical in basal and apical OHCs. A similar gating stiffness implies that the MET channel complex is identical in OHCs at the two cochlear locations (29) and disagrees with a previous report of a tonotopic gradient in single-channel gating stiffness (28).

### Effects of *Tmc1* Mutations on Channel Gating.

**Fig. 5.**
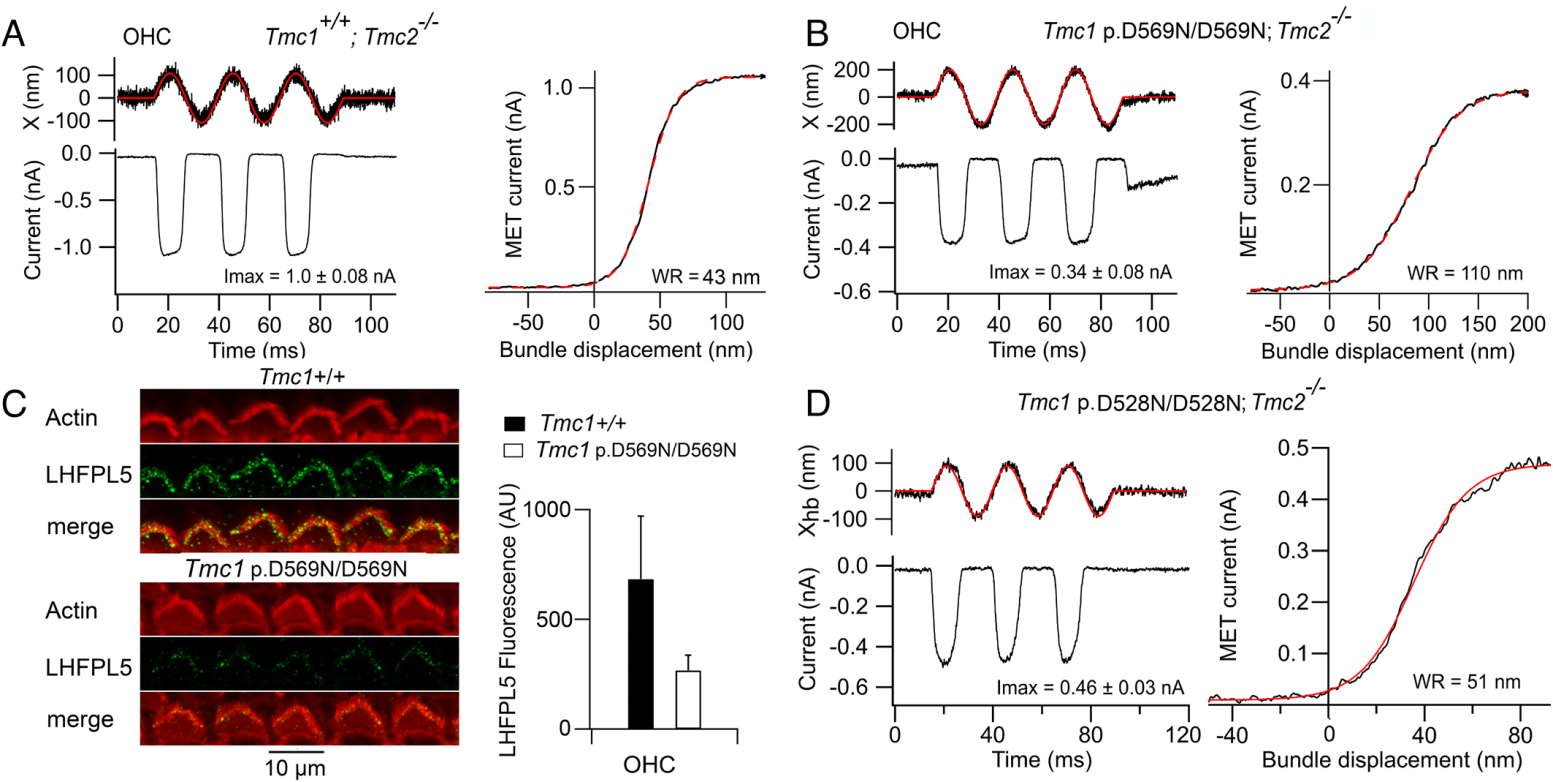
Effects of *Tmc1* mutations on the dynamic range of MET channel activation in apical OHCs. (*A*) MET currents in *Tmc1 +/+; Tmc2−/−* in response to fluid jet stimuli (*Left*), noisy trace is photodiode signal; transducer activation curve (*Right*) fit with Boltzmann equation with I_MAX_ = 1.06 nA, half-saturation X_O_ = 41 nm, and slope X_S_ = 9.8 nm, WR = 43 nm. (*B*) MET currents in *Tmc1* p.D569N/D569N; *Tmc2−/−* in response to fluid jet stimuli; transducer activation curve (*Right*) fit with Boltzmann equation with I_MAX_ = 0.37 nA, half-saturation X_O_ = 81 nm, and slope X_S_ = 25 nm, WR = 110 nm. Holding potential −84 mV. (*C*) Reduced LHFPL5 immunolabeling in *Tmc1* p.D569N/D569N. Loss of immunolabel in apical OHCs; *Right*, mean ± SD of LHFPL5 label indicates 62 percent loss of label. (*D*) MET currents in *Tmc1* p.D528N/D528N; *Tmc2−/−* for fluid jet stimuli; transducer activation curve (*Right*) fit with Boltzmann equation with I_MAX_ = 0.48 nA, half-saturation X_O_ = 37 nm and slope X_S_ = 11.6 nm, WR = 51 nm.

The site of interaction between LHFPL5 and TMC1 is not firmly established, but two regions have been suggested. LHFPL5 has four transmembrane domains, and the N-terminal half of LHFPL5 has been proposed to interact with a conserved amphipathic helix H3 (53) present in the N terminus of both TMC1 and TMC2 (21). An alternate site has been suggested based on single-molecule pull-down assays (20), which points to coupling between LHFPL5 and TMC1 partly involving TMC1 residues 559 to 578. The mutation *Tmc1* p.D569N (site of a human deafness mutation DFNA36) lies within this interaction range and reduces the maximum MET current (30), in the present experiments to 0.44 ± 0.19 nA (N = 7) (Fig. 5 *A* and *B*). The dominant *Tmc1* p.D569N mutation also reduces LHFPL5 immunolabeling in the bundle by over 60 percent (Fig. 5*C*). Like *Lhfpl5−/−*, the *Tmc1* p.D569N/D569N; *Tmc2−/−* mutation increased the width of the activation curve (Fig. 5 *A* and *B*): the mean WR was 98 ± 37 nm (N = 14) compared to 44 ± 6 nm (N = 7) for *Tmc1 +/+*; *Tmc2−/−*. However, the mean value for the mutant conceals a substantial spread, with some values grouped around the control and some at twice the control. Such a broad distribution was not seen with *Lhfpl5−/−*, which showed no results around the control value (Fig. 2*D*). The source of this variability is unknown, but it does not correlate with any other measured parameters, including the maximum current. The hair bundle stiffness in *Tmc1* p.D569N/D569N was determined as of 4.4 ± 2.9 mN/m (N = 7), and on perfusing with BAPTA, the stiffness was reduced to 4.2 ± 2.7 mN/m (N = 7). Tip links were still present (30) but their number, N_TL_, was reduced to 20 in proportion to the maximum current, and the geometrical factor γ increased to 0.2 (Table 1). The single-channel gating stiffness in *Tmc1* p.D569N/D569N was evaluated from Eq. **3** to be 0.28 ± 0.25 mN/m, comparable to that in *Lhfpl5−/−*. This is consistent with the D569N mutation interfering with the binding of LHFPL5 to TMC1 (20).

As a control for the effects on channel sensitivity in *Tmc1* missense mutations, we also examined the activation characteristics of two other mutants; the first was *Tmc1* p.D528N, which we previously demonstrated caused a 35 percent reduction in the single MET channel conductance (29, 31), consistent with the D528 residue occupying a site in the channel pore. Unlike the *Tmc1* p.D569N/D569N, the homozygous *Tmc1* p.D528N/D528N; *Tmc2−/−* had no effect on the width of the activation curve: the mean WR was 51 ± 5 nm (N = 5), not significantly different from 44 ± 6 nm (N = 7) for *Tmc1 +/+*; *Tmc2−/−* (*t* test, *P* = 0.5) (Fig. 5*D*). These measurements were performed on apical OHCs from P6 mice, with a maximum MET current of 0.46 ± 0.03 nA, and an apical OHC bundle height of 3.1 ± 0.3 µm (N = 13), both very similar to values in *Tmc1* p.D569N (0.44 ± 0.19 nA and 2.8 ± 0.2 µm). We also examined another missense mutation, *Tmc1* p.M412K/M412K; *Tmc2−/−* (*Beethoven*) (54), which had a WR of 53 ± 9 nm (N = 3), again not significantly different from *Tmc1+/+*; *Tmc2−/−.* We conclude that the broadening of the MET channel activation curve in *Tmc1* p.D569N was specific for that mutation, and there is no evidence from our I-X curves that the residue D528 in the sixth transmembrane domain (TM6) or M412 in TM4 is involved in gating. This conclusion disagrees with a previous claim that these mutations in TM4 and TM6 altered gating and reduced force sensitivity of the MET channel (38).

### Contribution of the Lipid Bilayer.

**Fig. 6.**
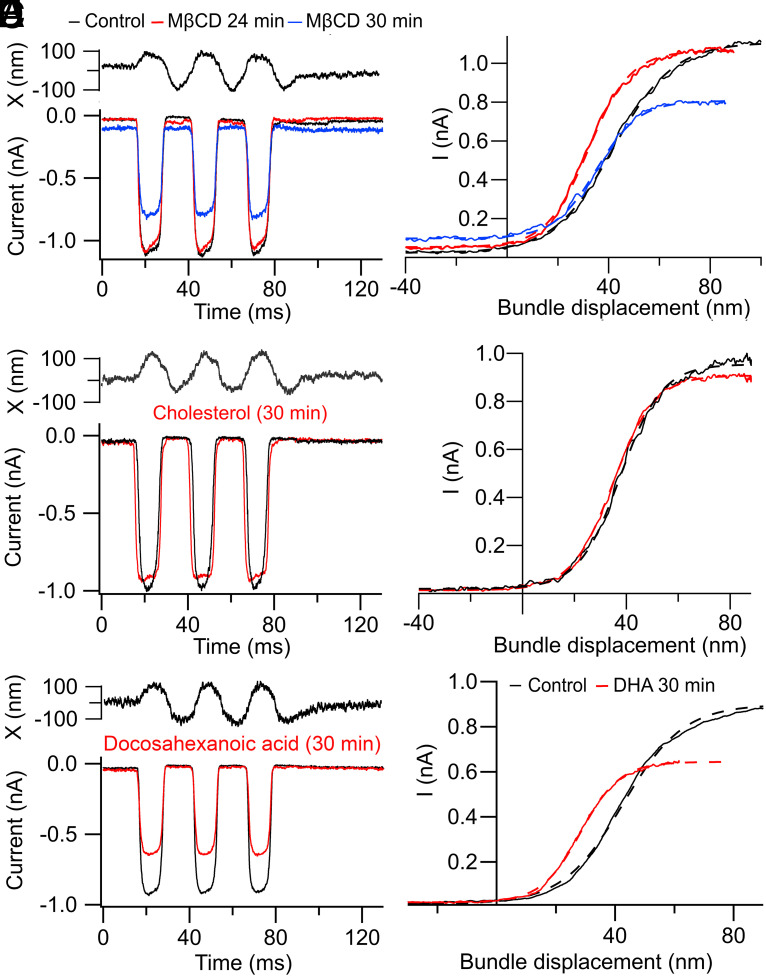
Effects of cholesterol in the lipid bilayer on MET channel activation. (*A*) MET currents in *Tmc1+/+*; *Tmc2+/+* during perfusion of 7 mM methyl-βcyclodextrin (MBCD) to deplete cholesterol. The three traces are the control (black) prior to introduction of MBCD, 24 min after the start of MBCD perfusion (red), and 30 min after beginning MBCD perfusion (blue). (*B*) MET current–displacement curves were fit with the Boltzmann equation with values of I_MAX_, half-saturation, X_O_, and WR: black, 1.09 nA, 42 nm, and 52 nm; red, 1.03 nA, 32 nm, and 36.5 nm; blue, 0.73 nA, 35 nm, and 36.5 nm. Note values for X_O_ and WR were both reduced, indicating enhanced sensitivity; apical OHCs from P6 mice. (*C*) MET currents in a P4 apical OHC of a *Tmc1+/+*; *Tmc2+/+* before and 30 min after starting perfusion of 7 mM water-soluble cholesterol to augment membrane cholesterol. Noisy traces at the top are the photodiode signals from the hair bundle. (*D*) Current–displacement relations for responses in *C*; curves fit with Boltzmann equation (dashed lines) with values of I_MAX_, half-saturation, X_O_, and WR of control (black): 0.98 nA, 38 nm, and 35 nm; cholesterol (red) 0.89 nA, 35 nm, and 35 nm, indicating little effect. (*E*) MET currents in a P5 apical OHC of a *Tmc1+/+*; *Tmc2+/+* before and 30 min after starting perfusion of 0.2 mM Na^+^ docosahexanoic (22:6) acid. (*F*) Current–displacement relations for responses in *E*; curves fit with Boltzmann equation (dashed lines) with values of I_MAX_, X_O_, and WR: control (black): 0.85 nA, 43 nm, and 45 nm; Na+ docosahexanoic acid (DHA) (red) 0.62 nA, 28 nm, and 29 nm. Note the increased transducer sensitivity with DHA was similar to that produced by depleting cholesterol. For all OHC recordings, holding potential −84 mV.

For most other mechanically sensitive ion channels, such as MscL and PIEZO1, it has been proposed that the mechanical force to open the channel is delivered through the lipid bilayer, a process referred to as force-from-lipid (4). Supporting evidence includes the ability to activate the channel when it is inserted into a reduced system composed only of lipid vesicles and that the channel behavior depends on the lipid composition of the bilayer (55, 56); for example, changing the amount of cholesterol influences channel gating possibly by altering the fluidity and thickness of the lipid bilayer (57). The proportion of cholesterol in the bilayer has been found to affect the force threshold for activation of the MscL mechanoreceptor channel (55). Cholesterol is a lipid constituent of the hair bundle membrane and can be localized with the fluorescent antibiotic filipin to the tips of the hair cell stereocilia, in the vicinity of the mechanotransduction apparatus (58). We therefore examined whether cholesterol manipulations could affect MET channel gating. Depletion of membrane cholesterol was achieved by perfusing the preparation with saline containing 7 mM methyl-β-cyclodextrin (MβCD), a cyclic glucose heptamer that sequesters the steroid. It took 20 min before any effects on the activation curve were visible, but there was then an increase in sensitivity and later a reduction in current amplitude (Fig. 6 *A* and *B*). The WR for transduction in OHCs of P6 *Lhfpl5* heterozygotes was significantly reduced from 48 ± 4 nm to 32 ± 4 nm (N = 3) (*t* test*, P* = 0.0007). Corroborative evidence of an increased sensitivity was the leftward shift in the half-saturation displacement, X_O_, from 42 ± 3 nm to 34 ± 2 nm. There was a subsequent reduction in MET current amplitude by 38 ± 12 percent from 0.88 nA (N = 6; *t* test*, P* = 0.003). This is larger than the expected rundown during a recording, which was less than 20 percent over 30 min. The enhanced sensitivity after cholesterol depletion may be partly attributable to an increased membrane fluidity affecting the motion of the components of the channel complex during activation. However, the run down in the current on cholesterol depletion argues that the lipid composition is set to optimize the MET current.

We also looked for evidence of an opposite desensitizing effect caused by increasing the membrane cholesterol with a water-soluble cholesterol (MβCD + cholesterol mixture) (59). Control measurements were taken at the start of the recording and then 7 mM of water-soluble cholesterol mixture was perfused. The cholesterol mixture made the bath solution very viscous, and 7 mM was the highest concentration achievable while still maintaining visibility of the cochlea. Although there was a small reduction in the MET current over the duration of the recording, no effect on the working range was evident after 30 min (Fig. 6 *C* and *D*). The mean current was reduced from 0.93 ± 0.06 nA (control) to 0.73 ± 0.15 nA 30 min after perfusion. The working range was 37 ± 4 nm (control) and 42 ± 7 nm (N = 4) after perfusion. The difference in working range is insignificant (*t* test*, P* = 0.45). One conclusion is that cholesterol is already present around the MET channel (58) and supplementary cholesterol has little effect, but removing cholesterol alters the membrane environment to facilitate conformation changes in the channel complex.

We also examined the effects on MET channel activation of Na^+^ docosahexanoic acid (DHA), a long-chain polyunsaturated fatty acid, shown to influence the gating of PIEZO1 (60) and to increase the charge movement in OHC prestin (61). The 22-carbon fatty acid could theoretically act by increasing bilayer thickness and fluidity. Perfusion of the fatty acid in three experiments boosted the sensitivity of MET channel activation, steepening the I-X curve [WR = 42 ± 6 nm (control) to 30 ± 5 nm (DHA)] and producing a negative shift in its half-activation [X_O_ = 59 ± 13 nm (control) to 35 ± 8 nm (DHA)] (Fig. 6 *E* and *F*). Both changes to the activation curve produced by DHA were statistically significant (*t* test*, P* < 0.005), and echo those of depleting cholesterol. In addition to the alterations in sensitivity, both manipulations resulted in long-term reduction in the maximum current, arguing that they destabilized the channel. Our results do not necessarily imply that force is delivered to the MET channel via the lipid bilayer, merely that movements of the protein complex during channel activation are influenced by the lipid composition of the bilayer.

## Discussion

The tetraspan protein LHFPL5 is one of three known components, along with TMIE and TMC1 (and/or TMC2), of the hair cell MET channel complex. TMC1 forms the ion channel (11), and the complex is thought to be anchored extracellularly by the tip link PCDH15 (12, 13) and intracellularly by CIB2 (15, 16, 62). LHFPL5 was previously believed necessary in mice to correctly localize TMC1 to the transduction site at the tips of the stereocilia, but it is not obligatory for forming an ion channel (17). In its absence, the amount of TMC1/2 in the stereocilia is diminished but the MET current, although reduced, is still measurable at 200 to 400 pA in neonatal OHCs of *Lhfpl5* knockouts (Fig. 2*C*). In zebrafish, localization of TMC1 does not depend upon *Lhfpl5* in either the inner ear or lateral line organ, and in *Lhfpl5* mutants, Tmc1 and Tmc2b proteins still localize to the hair cell stereocilia (63). The reduction in MET current in *Lhfpl5* knockout may be attributable to stabilization of the MET complex by LHFPL5 and largely account for the autosomal recessive nonsyndromic hearing loss DFNB66/67 (23, 64). In searching for another role, we suggest that LHFPL5 is part of the linkage apparatus for transmitting force to activate the MET channel. In *Lhfpl5* knockouts, the channel gating sensitivity (Z) was diminished several-fold (Fig. 2*B*), and the stiffness of the channel’s gating spring was virtually abolished (Fig. 4). The gating stiffness per tip link, κ_GS_, was reduced over an order of magnitude from 2.2 mN/m to 0.2 mN/m. Each tip link comprises two PCHD15 molecules (19, 65), the C termini of which deliver force to two channel complexes, so the single-channel gating stiffness is half those values measured, 1.1 mN/m per channel in the wild type. The present and previous results (21, 22, 29) indicate that LHFPL5 augments the sensitivity of transduction by connecting PCDH15 to TMC1.

According to the gating spring model of MET channel activation, the single-channel gating force Z and the single-channel gating stiffness, κ_GS_, are related by Z = γ* κ_GS_*d, where d is the distance that the spring shortens on channel opening (26). From our experimental values for Z (344 fN), γ (0.14), and κ_GS_ (2.2 mN/m) in *Tmc1+/+; Lhfpl5+/−* mice (equivalent to wild type), d is evaluated as 1.1 nm. This seems a reasonable movement for a conformational change in a protein. However, using the altered values in the *Lhfpl5−/−* experiments of Z (130 fN), γ (0.18), and κ_GS_ (0.2 mN/m), d becomes 4 × 10^−3^ nm, which seems too small for the same channel opening, and implies κ_GS_ for the *Lhfpl5* knockout has been underestimated. To comply with an unchanged d of 1.1 nm, assuming the same channel motion in the *Lhfpl5* knockout, κ_GS_ would need to be 0.66 mN/m, a third of wild type, if Z and γ are accurate. Similar calculations can be performed on *Tmc1* p.D569N where LHFPL5 is down-regulated and κ_GS_ is small. There are two arguments to account for the discrepancy. One is that the relationship between Z and κ_GS_ is complex, possibly due to non-Hookean behavior of the gating spring. Another is that the action of BAPTA does more than sever the tip links and has a secondary effect to increase hair bundle pivotal stiffness. If the latter occurred, κ_GS_ would be underestimated in both control and *Lhfpl5* mutants. The source of the discrepancy is currently unclear.

An important related question pertains to how the channel is gated in the absence of LHFPL5. There are two possible routes, one by a direct connection from PCDH15 to TMC1 and the other via an indirect transmission through the lipid from PCDH15 whose C terminus traverses the bilayer. Interactions between PCDH15 and both TMC1 and TMC2 have been described in both mouse and zebrafish (17, 18). In zebrafish, the interaction with PCDH15 may involve the N terminus of Tmc2a (18). TMIE is a possible intermediary, as it interacts with the PCDH15-CD2 isoform (66), is a part of the TMC1-based channel (53), and contributes to channel conductance (29). The MET channel could still be gated in the absence of TMIE (29), though the currents were too small to quantify the WR. The possibility of force transmission via the lipid bilayer has been examined for several mechano-sensitive channels, especially the bacterial MscL where the effects of cholesterol were documented (55). The effects of membrane cholesterol have also been demonstrated to boost the voltage-dependent gating of prestin in OHCs (67). The main processes underlying the action of cholesterol and docosahexanoic acid on channel gating are changes in the fluidity or thickness of the membrane bilayer. It must be emphasized that altering MET channel activation by either agent is not primary evidence that force from PCDH15 is delivered to the channel along the bilayer. Moreover, for channels such as MscL that respond to cell swelling, the mechanosensitive channel is stimulated by lateral forces in the plane of the bilayer. In contrast, stimulation of the hair cell MET channel occurs by tip-link force applied perpendicular to the plane of the bilayer.

## Data Availability

Data have been deposited in a publicly accessible database with DOI: https://doi.org/10.5061/dryad.0vt4b8h5r (68).
